# Impacts of Environmental Concentrations of Nanoplastics on Zebrafish Neurobehavior and Reproductive Toxicity

**DOI:** 10.3390/toxics12080617

**Published:** 2024-08-21

**Authors:** Ziqing Sun, Baihui Wu, Jia Yi, Haiyang Yu, Jiaxuan He, Fei Teng, Tong Xi, Jinlong Zhao, Jing Ruan, Peiye Xu, Runchao Tao, Liushuo Jia, Hao Ji

**Affiliations:** 1 Institute of Metal Research, Chinese Academy of Sciences, 72 Wenhua Road, Shenyang 110016, China; 2 Institute of Life Science & Biomedical Collaborative Innovation Center of Zhejiang Province, Wenzhou University, Wenzhou 325035, China; 3 Civil Aviation College, Shenyang Aerospace University, Shenyang 110136, China; 4 College of Animal Science and Veterinary Medicine, Shenyang Agricultural University, Shenyang 110866, China

**Keywords:** environmental pollutants, polystyrene nanoplastics, reproductive and neurobehavioral effects, mechanism, oxidative stress

## Abstract

Nanoplastics, as emerging environmental pollutants, can transport contaminants across marine environments, polluting pristine ecosystems and being ingested by marine organisms. This transfer poses a severe threat to global aquatic ecosystems and potentially impacts human health through the food chain. Neurobehavioral and reproductive toxicity are critical areas of concern because they directly affect the survival, health, and population dynamics of aquatic species, which can have cascading effects on the entire ecosystem. Using zebrafish as a model organism, we investigated the toxic effects of environmental concentrations of polystyrene nanoplastics (PS-NPs). Behavioral assessments, including the novel tank test and open field test, demonstrated significant neurobehavioral changes, indicating increased anxiety and depressive behaviors. A pathological analysis of brain and gonadal tissues, along with evaluations of neurobehavioral and reproductive toxicity biomarkers, revealed that exposure to PS-NPs leads to brain tissue lesions, inflammatory responses, oxidative stress activation, hormone level disruptions, and gonadal damage. Real-time quantitative PCR studies of reproductive gene expression further showed that PS-NPs disrupt the endocrine regulation pathways of the brain-pituitary-gonadal (BPG) axis, causing reproductive toxicity with sex-specific differences. These findings provide crucial insights into the impacts of nanoplastics on aquatic organisms and their ecological risks, offering theoretical support for future environmental protection and pollutant management efforts.

## 1. Introduction

Since 1950, the production and use of plastics has increased significantly. According to the data, more than 400 million tons of plastic were produced on Earth in 2022 [1]. The majority of marine microplastic pollution originates from land, with an estimated 4.8–12.7 metric tons of plastics entering marine ecosystems each year [2]. Plastic debris varies in type and size, with low-density polyolefins and polystyrene being the most abundant nanoplastics found in seawater. Plastics larger than 5 mm are classified as microplastics, while those smaller than 5 mm are termed nanoplastics (NPs) [3,4]. Unlike macroplastics, which are visible and easily captured, NPs are characterized by their small size, hydrophobic nature, and resistance to degradation, making them prone to bioaccumulation in aquatic organisms [5].

Increasing evidence highlights the toxic effects of micro- and nanoplastics (MNPs) on various organisms, including developmental, neurotoxic, reproductive, immunotoxic, and genotoxic impacts, as well as transgenerational effects [6,7]. Reproduction is vital for maintaining the balance of aquatic ecosystems, ensuring species population and contributing to the stability and health of ecosystems. The reproductive system is one of the most vulnerable biological systems, and studies have shown that NPs can impair the development of multiple organs, especially reproductive organs, thereby affecting fertility [7,8]. For example, research on *Ceriodaphnia dubia* demonstrated that acute and chronic exposure to polyester fibers significantly affected reproduction and survival [9]. Similarly, exposure to MNPs impaired the reproductive capacity of oysters, reducing egg production, sperm vitality, and offspring growth [10].

In fish, NPs have been associated with adverse effects such as hepatic stress, intestinal damage, oxidative stress, and neurobehavioral changes. NPs have also been detected in various tissues of terrestrial organisms, including humans, such as the brain, blood, placenta, gonads, and semen [11]. The detection of MNPs in fetal chambers and the growing concern over infertility underscore the urgency of studying the reproductive toxicity and mechanisms of NPs. Zebrafish (*Danio rerio*) are widely used as a model organism for reproductive studies due to their high genetic similarity to humans, with 87% gene homology [12]. Zebrafish share similar physiological structures, signaling pathways, and functions with mammals, making them suitable for evaluating drug toxicity, reproductive toxicity, and environmental pollutants, including NPs [13]. Zebrafish exhibit a diverse range of behaviors, including social interactions, exploration, foraging, and reproduction, which integrate responses to internal and external stimuli. This makes zebrafish embryos, larvae, and adults ideal for in vivo and in vitro toxicological studies [14].

Previous studies have indicated that exposure to high concentrations of PS-NPs significantly increases reactive oxygen species (ROS) levels in zebrafish gonadal tissues [15]. NPs can penetrate the blood–brain barrier of zebrafish, leading to alterations in brain tissue integrity, acetylcholinesterase activity, neurotransmitter levels, and oxidative stress, potentially resulting in behavioral changes and movement disorders [16]. However, the mechanisms underlying the combined neurobehavioral and reproductive toxicity of NPs remain unclear and require further investigation.

**Figure 1:**
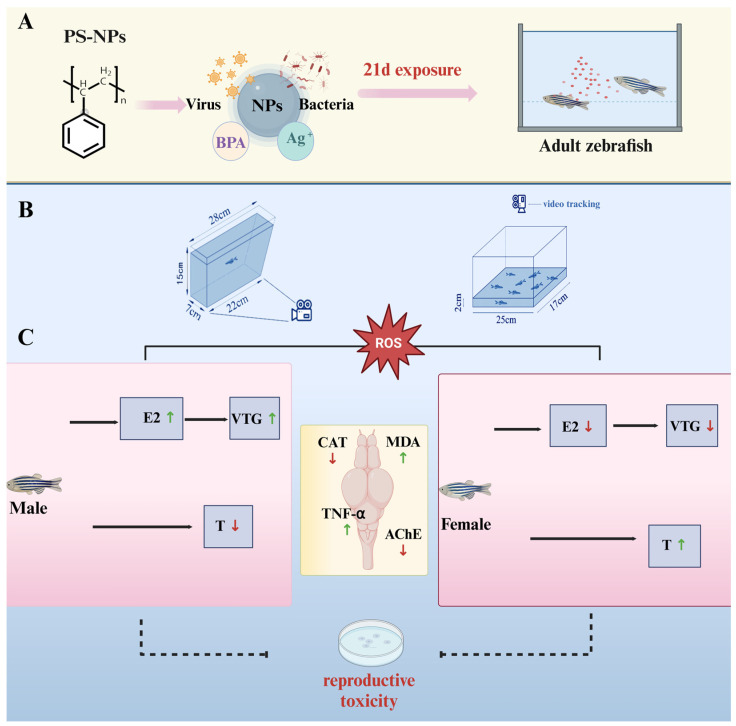
Experimental process. (**A**) Zebrafish exposed to PS-NPS for 21 days; (**B**) Two experimental devices are used to study the behavior change in zebrafish; (**C**) Effects of PS-NPs exposures on the reproductive gene expression in zebrafish (the results are based on our experimental research). PS-NPs, Polystyrene nanoplastics; NPs, Nanoplastics; BPA, Bisphenol A; E2, Estradiol; VTG, Vitellogenin; T, Testosterone; CAT, Catalase; MDA, Malondialdehyde; TNF-α, Tumor necrosis fac-tor-alpha; AChE, Acetylcholinesterase.

The aim of this study was to investigate the interaction between neurobehavioral toxicity and reproductive toxicity in adult zebrafish exposed to environmentally relevant concentrations of nanoplastics. We evaluated a variety of biomarkers, including behavioral changes, brain and gonad pathology, oxidative stress levels, hormone levels, and gene expression in the brain–pituitary–gonad (BPG) axis [17,18,19,20,21], to shed more light on the potential effects of polystyrene nanoparticles (PS-NPs) on the nervous and reproductive systems (Figure 1). The findings provide new insights into the complex mechanisms between neurobehavioral toxicity and reproductive toxicity of nanoplastics, highlight their potential risks to human health, and provide an important scientific basis for environmental protection and pollutant management.

## 2. Materials and Methods

### 2.1. Experimental Animals and Reagents

#### 2.1.1. Experimental Animals

Adult zebrafish, aged 6–7 months, were obtained from Shanghai Yino Biotechnology Co., Ltd. (Shanghai, China). The fish were maintained in a zebrafish recirculating aquaculture system under standard laboratory conditions. Artemia cysts, used as fish feed, were purchased from Tianjin Fengnian Aquaculture Co., Ltd. (Tianjin, China) and stored at −20 °C until use.

#### 2.1.2. Reagents

PS-NPs with a diameter of 50 nm were procured from Xi’an Qiyue Biotechnology Co., Ltd. (Xi’an, China). The following enzyme-linked immunosorbent assay (ELISA) kits were used: catalase (CAT), malondialdehyde (MDA), vitellogenin (VTG), tumor necrosis factor-alpha (TNF-α), acetylcholinesterase (AChE), testosterone (T), and estradiol (E2). These kits were purchased from Wenzhou Yino Medical Equipment Co., Ltd. (Wenzhou, China). Other reagents included the gDNA Remover Kit (Wenzhou Yino Medical Equipment Co., Ltd.), TRIzol reagent (Life Technologies, Shanghai, China), PrimeScript RT Reagent Kit, and SYBR Green PCR Kit (TaKaRa, Tokyo, Japan). The above kits were purchased from Wenzhou Yino Medical Equipment Co., Ltd. (Wenzhou, China).

### 2.2. Zebrafish Husbandry and Experimental Design

In this study, a threshold concentration of 1 mg/L was established to accurately assess the potential neurotoxic and reproductive effects of nanoplastics on aquatic animals in the environment. In numerous laboratory studies investigating the toxicity of nanoplastics in the environment, a common practice was to use a concentration of 1 mg/L as the threshold for exposure to nanoplastics [22].

Zebrafish (both sexes) were housed in 12 L transparent glass tanks at a density of 5 fish/L. The tanks were maintained at 28.0 ± 2 °C with aerated water (pH: 6.9–7.2, dissolved oxygen: 6.0–8.0 mg/L, conductivity: 480–510 μS/cm, hardness: 53.7–71.6 mg/L CaCO_3_) under a 14:10-h light–dark cycle. Fish were fed Artemia twice daily [23].

The experiment consisted of two groups: a control group and a PS-NPs’ exposure group. Each group comprised 20 fish. The PS-NPs’ concentration was set at 1 mg/L. Prior to exposure, fish were acclimated for 7 days. The exposure period lasted 21 days, during which the water was changed every 72 h. For each water change, 240 μL of a PS-NPs stock solution (50 nm, 50 mg/mL) was added to 12 L of water to maintain a consistent exposure concentration. No mortality or abnormal behavior was observed during the exposure period. All procedures were conducted in accordance with the guidelines of the China Food and Drug Administration for the care and use of laboratory animals. The Committee for Animal Experiments of Wenzhou University approved all the protocols and procedures for experiments involving zebrafish (No. WZU-2024-058), and all experiments were conducted in accordance with the guidelines for experimental animals.

Before sampling, fish were fasted for 24 h and rinsed with saline to remove external nanoplastics. Fish were anesthetized on ice, and their wet body weights were measured using an electronic balance. Brain and gonadal tissues were collected, weighed, and pooled in 1.5 mL centrifuge tubes (*n* = 4 per pool) for homogenate preparation.

#### 2.2.1. Behavioral Tracking and Quantification

After 21 days of exposure, zebrafish behavior was assessed using two tests: the novel tank test (NTT) and the open field test (OFT). The experimental setup included rectangular glass aquaria (25 × 17 × 15 cm; L × W × D) filled to a height of 2 cm with water. Individual fish were placed at the bottom of the tank and their swimming behavior was recorded for 10 min using an infrared HD camera mounted above the tank. The first 10 min of recording served as an acclimation period, followed by three parallel trials (*n* = 20 fish per group) [24,25].

The videos were analyzed using FishTrack zebrafish behavior analysis software (Shanghai Xinsoft, version 2.0 Copyright © 2019). The software automatically tracked swimming speed, behavior intensity, and movement patterns. A virtual grid divided the tank into upper and lower zones, and parameters such as the total distance moved (cm), average speed (cm/s), latency to enter the upper zone (s), number of entries to the upper zone, and number of entries to the lower zone were measured to assess anxiety-like behavior. Each trial lasted 5 min, with *n* = 6 fish per group. After behavioral assessments, fish were euthanized, and brain, liver, and muscle tissues were dissected for biochemical measurements. 

#### 2.2.2. Histopathology and Ultrastructure of Brain and Gonadal Tissues

Fresh tissue samples were fixed in Bouin’s solution for 24 h. Gonadal tissues were dehydrated using a graded ethanol series (75%, 85%, 95%, 100%), cleared in xylene, embedded in paraffin, sectioned, stained with hematoxylin and eosin (HE), and mounted with neutral resin. Tissue sections (the thickness of the sections in this experiment was 3–5 µm) were observed and imaged under a Nikon Eclipse Ci-L microscope (Tokyo, Japan).

#### 2.2.3. Assessment of Oxidative Stress Parameters and Biochemical Indices

Fresh tissue samples were dissected, immediately frozen in liquid nitrogen, and stored at −80 °C for the assessment of oxidative stress parameters and biochemical indices. Brain homogenates were analyzed for CAT and MDA levels using ELISA kits. Four fish were pooled per replicate, with five replicates per assay. Gonadal homogenates were analyzed for VTG, T, and E2 levels. Eight fish were pooled per replicate, with three replicates per assay. The procedures followed the manufacturer’s instructions, and standard curves provided by the kits were used for quantification (R^2^ > 0.99).

#### 2.2.4. RT-qPCR

Zebrafish exposed to PS-NPs (1 mg/L, 50 nm) for 21 days were analyzed for HPG axis-related gene expression using RT-qPCR. Beta-actin was used as the housekeeping gene. Total RNA was extracted from fresh gonadal tissues using the TRIzol reagent. Complementary DNA (cDNA) was synthesized using a reverse transcription kit. RT-qPCR was performed using the SYBR Green Master Mix on a QuantStudio 6 Flex real-time PCR system. The reaction conditions were as follows: 95 °C for 30 s, followed by 40 cycles of 95 °C for 5 s and 60 °C for 34 s. Gene expression was analyzed using the 2^−ΔΔCT^ method, with beta-actin as the reference gene. The primers for the target genes, involved in steroid hormone synthesis, were verified using NCBI Primer-BLAST and are listed in Table 1.

### 2.3. Statistical Analysis

Experimental data were expressed as the mean ± standard error. The statistical analysis and graphical representation were performed using GraphPad Prism 9 software. A one-way analysis of variance (ANOVA) was used for multiple group comparisons, and *t*-tests were used for comparisons between two groups. Significance levels were set at *p* > 0.05 (ns), *p* < 0.05 (*), *p* < 0.01 (**), *p* < 0.001 (***), and *p* < 0.0001 (****).

## 3. Results

### 3.1. Neurobehavioral Changes in Zebrafish Induced by PS-NPs’ Exposure

#### 3.1.1. Increased Bottom-Dwelling Behavior in Zebrafish

**Figure 2:**
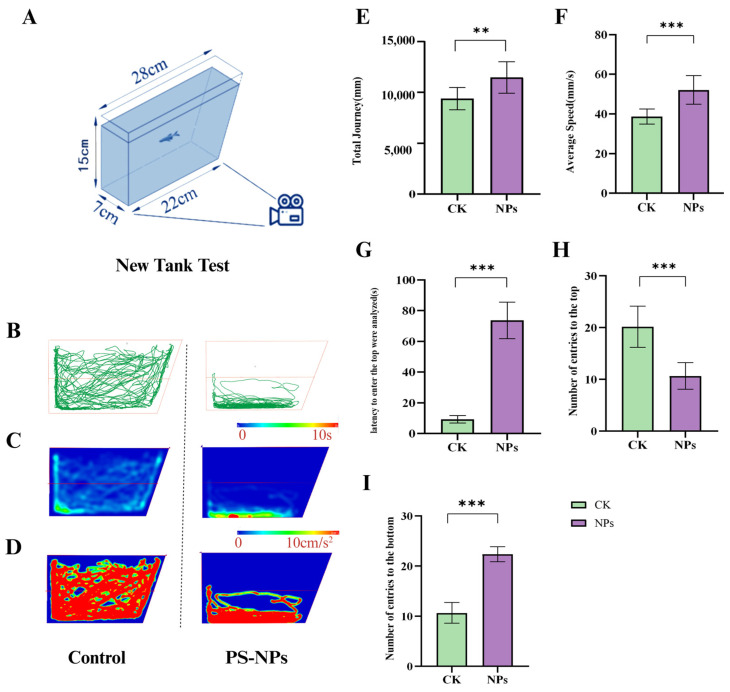
Anxiety-like behavior of zebrafish in the novel tank test. (**A**) Schematic diagram of NTT; (**B**) zebrafish motion track diagram; (**C**) activity track heat map; (**D**) 2D activity map; (**E**) total Journey (mm); (**F**) average speed (mm/s); and (**G**) latency to enter the top (s) were analyzed (s); (**H**) number of entries to the top; (**I**) number of entries to the bottom. Numbers of zebrafish: NTT, *n* = 10. Error bars represent standard deviation (SD). (* *p*  <  0.05, ** *p*  <  0.01, and *** *p*  <  0.001 indicates a statistically significant difference compared to the control group). PS-NPs, polystyrene nanoplastics; NPs, Nanoplastics.

After 21 days of exposure, behavioral endpoints in the novel tank test (NTT) were compared between the control group and PS-NPs-exposed group. The results indicated that zebrafish exposed to PS-NPs exhibited significantly increased swimming speed, total distance traveled, and latency to enter the top of the tank compared to the control group (Figure 2E–G); there are some differences between PS-NPs in terms of total journey and the PS-NPs group was significantly different from the control group. Additionally, there was a marked increase in bottom-dwelling behavior in the PS-NPs-exposed fish. This shift in swimming and activity patterns suggests a behavioral response to the presence of nanoplastics. The statistical analysis showed significant differences in the preference for swimming zones (upper vs. lower) between the two groups. The PS-NPs-exposed zebrafish displayed more unstable movement and reduced exploratory behavior. The increased latency to enter the upper zone and the strong preference for the bottom zone observed in the exposed group, but not in the control group, are indicative of heightened anxiety-like behavior (Figure 2B–D, Videos S1 and S2; Figure 2H,I), and the PS-NPs group was significantly different from the control group.

#### 3.1.2. Decreased Average Speed and Exploratory Behavior

**Figure 3:**
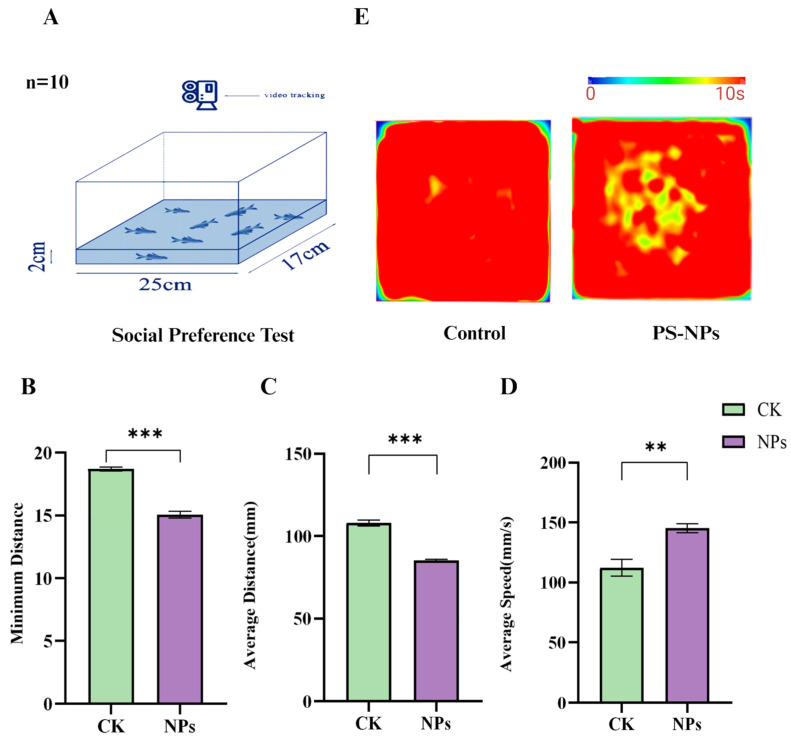
Social behavior experiment in zebrafish. (**A**) Schematic diagram of the open field test; (**B**) minimum distance (mm); (**C**) average distance (mm); (**D**) average speed (mm/s); (**E**) activity track heat map. Numbers of zebrafish: NTT, *n* = 20, 10 per group. Error bars represent standard deviation (SD). (* *p*  <  0.05, ** *p*  <  0.01, and *** *p*  <  0.001 indicates a statistically significant difference compared to the control group). PS-NPs, polystyrene nanoplastics; NPs, aanoplastics.

The open field test (OFT) further demonstrated significant behavioral differences between the PS-NPs-exposed group and the control group. The minimum distance moved and the average distance traveled by the PS-NPs-exposed zebrafish were significantly reduced, while their average swimming speed was markedly increased (Figure 3B–D; there are significant differences in minimum distance and average distance between the PS-NPs group and control group, and there are differences in average speed. This increased speed could be an active physiological response to stress. Heatmap analyses of swimming trajectories revealed a strong preference for the central area in the PS-NPs-exposed fish, suggesting neurological or behavioral abnormalities, whereas the control group showed no such preference. Evaluating zebrafish movement towards the top of the tank and their exploratory behavior, along with group activity behavior, helped quantify their anxiety-like response to the new environment, a common method for assessing animal depression models. The heatmap trajectories in the OFT confirmed that the PS-NPs-exposed zebrafish experienced environmental stress, leading to tighter clustering behavior as they sought safety. Consequently, PS-NPs’ exposure reduced exploratory behavior and induced anxiety-like and depressive behaviors in zebrafish (Figure 3E, Videos S1 and S2).

### 3.2. PS-NPs Induced Changes in Zebrafish Brain and Gonadal Tissues

**Figure 4:**
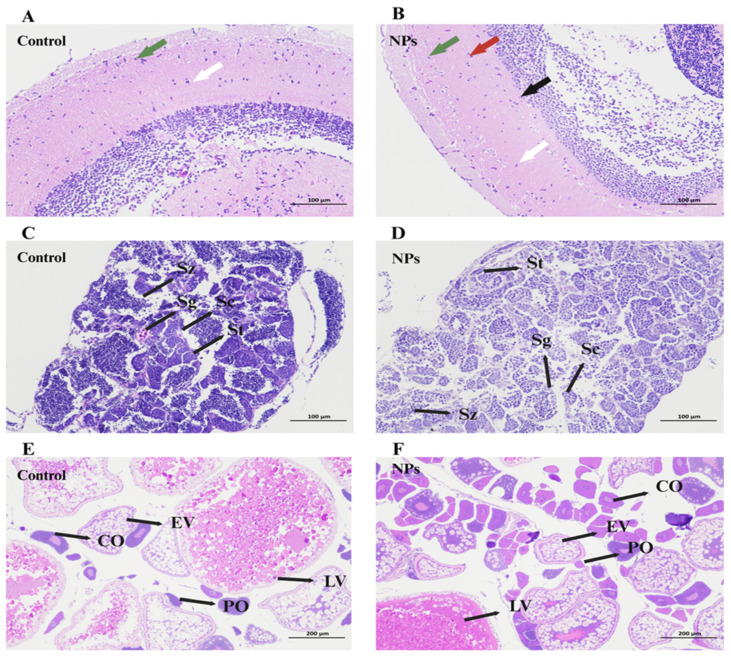
Histomorphometric analysis of organization from zebrafish exposed to 1 mg/L PS-NPs. (**A**,**C**,**E**) Control; (**B**) brain of zebrafish after PS-NPs exposure (HE × 200); (**D**) Testis histology of male zebrafish after PS-NPs’ exposure (HE × 200); (**F**) ovaries histology of female zebrafish after PS-NPs’ exposure (HE × 100). Sz, spermatozoa; Sg, spermatogonia; Sc, spermatocytes; St, spermatids; CO, corticolar alveolar oocytes; EV, early vitellogenin oocytes; LV, late vitellogenin oocytes; PO, perinuclear oocytes. The arrows in figures (**A**,**B**) indicate neurons.

In the PS-NPs-exposed group, zebrafish brain tissue exhibited vacuolar degeneration in the neurons. This was characterized by the presence of vacuolated neurons (indicated by green arrows), neurons with central chromatolysis (black-red arrows), and true basophilic necrotic neurons (black arrows). Acute eosinophilic necrosis of neurons was also observed (white arrows in Figure 4B), in contrast to relatively normal neurons in the control group (white arrows in Figure 4A).

In the testes of male zebrafish, various stages of spermatogenesis were observed, including spermatogonia (Sg), spermatocytes (Sc), spermatids (St), and spermatozoa (Sz) (Figure 4B,C). After 21 days of exposure, the control group showed a rich distribution of sperm cells, with a uniform distribution of spermatocytes, spermatogonia, and spermatids (Figure 4C). In contrast, PS-NPs exposure resulted in numerous vacuoles in the testes, with a significant reduction in the number of mature spermatids (St) and spermatozoa (Sz), and an increase in the number of spermatogonia (Sg) and spermatocytes (Sc) (Figure 4D). This could be attributed to the accumulation of NPs in the testes.

The development stages of oocytes in the ovaries included perinucleolar oocytes (POs), cortical alveolar oocytes (COs), early vitellogenic oocytes (EVs), and late vitellogenic oocytes (LVs) (Figure 4E). Exposure to PS-NPs resulted in a decrease in the number of secondary follicles and an increase in the number of primary follicles, with mature oocytes being smaller than those in the control group. Additionally, PS-NPs exposure led to the vacuolization of the oocytes and disrupted the connections between the oocytes and follicular cell layers (Figure 4F). This indicates that NP accumulation in the ovaries inhibited the development and maturation of oocytes.

### 3.3. NPs-Induced Changes in Oxidative Stress and Sex Hormone Levels in Zebrafish

Exposure to polystyrene nanoplastics (PS-NPs) significantly altered oxidative stress markers and sex hormone levels in zebrafish.

**Figure 5:**
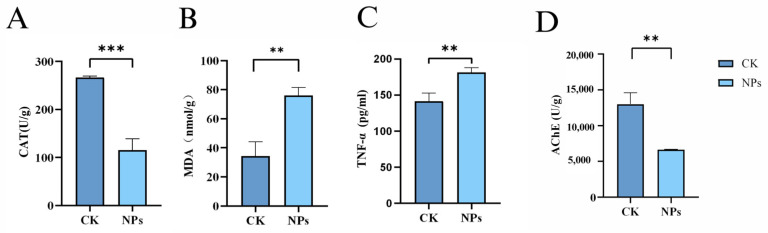
Effects of PS-NPs’ exposures on the oxidative stress and tumor necrosis factor-α in zebrafish. (**A**) CAT concentration; (**B**) MDA concentration; (**C**) TNF-α concentration; (**D**) AChE concentration. *n* = 20, 4 per group. Error bars represent standard deviation (SD). (* *p*  <  0.05, ** *p*  <  0.01, and *** *p*  <  0.001 indicates a statistically significant difference compared to the control group). NPs, Nanoplastics; CAT, Catalase; MDA, Malondialdehyde; TNF-α, Tumor necrosis fac-tor-alpha; AChE, Acetylcholinesterase.

Figure 5A,B show that the concentration of CAT significantly decreased while MDA levels markedly increased following 21 days of exposure to environmental concentrations of PS-NPs (1 mg/L, 50 nm). Additionally, Figure 5C indicates an increase in TNF-α levels in the brain, and Figure 5D shows a substantial decrease in AChE activity. The results showed that there were significant differences in CAT concentration between the PS-NPs group and control group, but there were differences in MDA, TNF-α and AChE concentration.

**Figure 6:**
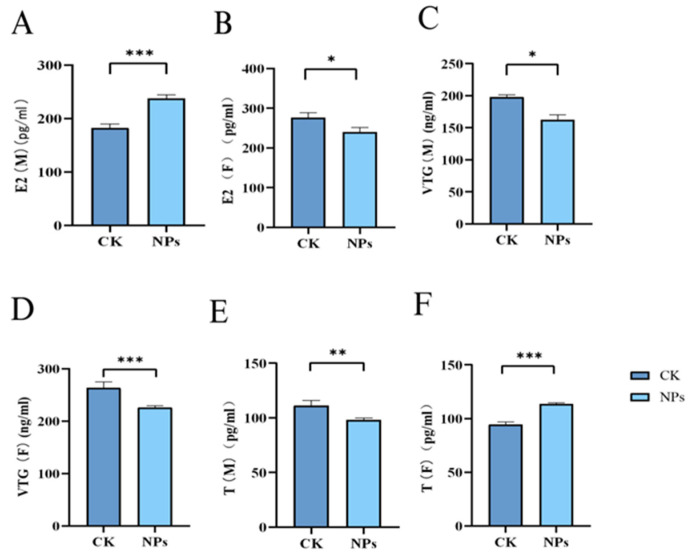
Effects of PS-NPs’ exposures on the sex hormones and vitellogenin in zebrafish. (**A**) E2 concentration (male); (**B**) E2 concentration (female); (**C**) VTG concentration (male); (**D**) VTG concentration (female); (**E**) T concentration (male); (**F**) T concentration (female). *n* = 20, 6 or 7 per group (equivalent tissue). Error bars represent standard deviation (SD). (* *p*  <  0.05, ** *p*  <  0.01, and *** *p*  <  0.001 indicates a statistically significant difference compared to the control group). NPs, Nanoplastics; E2, Estradiol; VTG, Vitellogenin; T, Testosterone; M, Male; F, Female.

Many pollutants disrupt the endocrine system and inhibit hormone production, affecting gonadal and gamete development. The homeostasis of sex hormones is crucial for the normal functioning of the endocrine system, and changes in sex hormone levels are commonly used to assess endocrine disruption. As shown in Figure 6A–F, exposure to NPs inhibited the secretion of E2 and VTG in female zebrafish, while increasing T levels. In male zebrafish, E2 and VTG levels increased while T levels decreased compared to the control group. This suggests that NPs’ exposure disrupted the balance of testosterone and estradiol, potentially converting testosterone into estradiol and leading to decreased testosterone and increased estradiol levels. In female zebrafish, the reduction in E2 and increase in T might result from inhibited follicular development and prolonged follicle maturation. The experimental results showed that compared with the control group, the E2 level in male fish in the PS-NPs group was significantly increased, while there was a certain difference in female fish. At the same time, the experimental results showed that compared with the control group, the VTG level in female fish in the PS-NPs group was significantly increased, while there was a certain difference in male fish. Compared with the control group, there were significant differences in T levels between male and female fish in the PS-NPs group.

### 3.4. NPs-Induced Changes in Reproductive Gene Expression in Zebrafish

**Figure 7:**
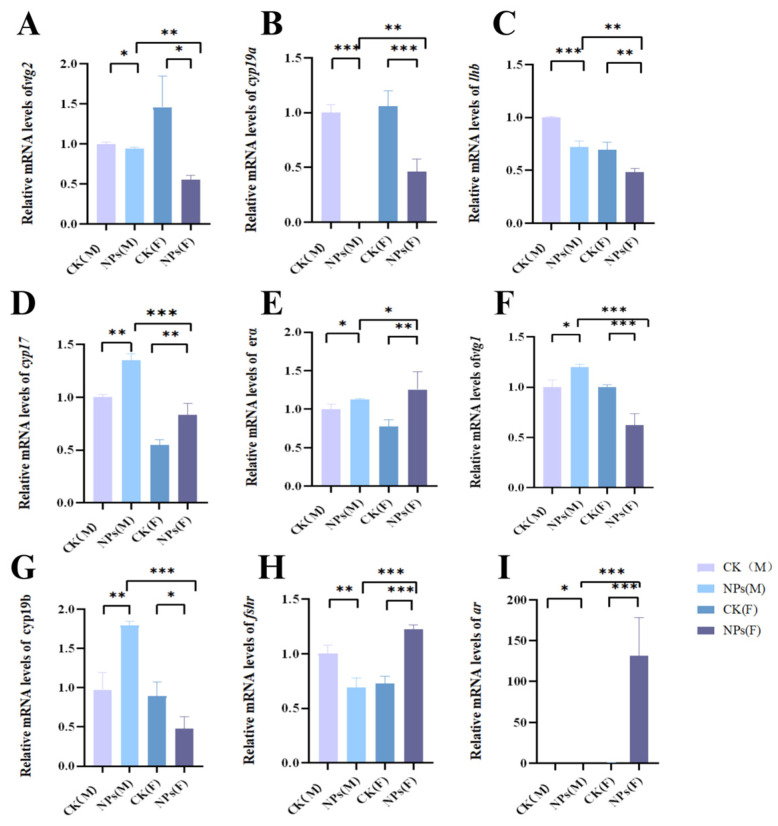
Effects of PS-NPs’ exposures on the reproductive gene expression in zebrafish. (**A**) Relative mRNA levels of *vtg2*; (**B**) relative mRNA levels of *cyp19a*; (**C**) relative mRNA levels of *lhb*; (**D**) relative mRNA levels of *cyp17*; (**E**) relative mRNA levels of *erα*; (**F**) relative mRNA levels of *vtg1*; (**G**) relative mRNA levels of *cyp19b*; (**H**) relative mRNA levels of *fshr*; (**I**) telative mRNA levels of *ar*. Error bars represent standard deviation (SD). (In the figure, M represents male fish and F represents female fish; * *p*  <  0.05, ** *p*  <  0.01, and *** *p*  <  0.001 indicates a statistically significant difference compared to the control group). NPs, Nanoplastics; *vtg2*, Vitellogenin 2; *cyp19a*, Cytochrome P450 19A; *lhb*, Luteinizing hormone beta; *cyp17*, Cytochrome P450 17; *erα*, Estrogen receptor alpha; *vtg1*, Vitellogenin 1; *cyp19b*, Cytochrome P450 19B; *fshr*, Follicle-stimulating hormone receptor; *ar*, Androgen receptor.

Exposure to PS-NPs led to notable alterations in the expression of several reproductive genes in both male and female zebrafish. Specifically, the expression of *vtg2*, *cyp19a*, and *1hb* genes was downregulated, while *cyp17* and *era* genes were upregulated in both sexes. In male zebrafish, *vtg1* and *cyp19b* expression levels increased, whereas these genes were downregulated in females. Additionally, *fshr* and *ar* were downregulated in males but upregulated in females (Figure 7A–I).

The BPG axis is crucial for regulating the production of sex hormones such as E2 and T in fish. Our study found that microplastic exposure significantly modulated the BPG axis in females, leading to the downregulation of genes involved in steroidogenesis. This resulted in decreased plasma levels of E2 and increased T levels, causing endocrine disruption. We hypothesize that NPs interfere with the reproductive process by affecting sex hormone secretion and altering BPG axis gene expression [26,27].

Exposure to PS-NPs for 21 days led to decreased mRNA expression levels of *cyp19a* and *cyp19b* and increased *cyp17* mRNA expression in female zebrafish. During this period, E2 levels decreased, and T levels increased. The cyp19a mRNA is primarily expressed in the ovaries and plays a role in converting testosterone to estrogen. Its expression is influenced by the demand for estrogen in various tissues, including the brain, retina, pituitary, and ovaries. The *cyp17* gene is a key regulatory gene in testosterone synthesis, converting cholesterol to testosterone. We speculate that PS-NPs induce increased T levels in female zebrafish, disrupting sex hormone homeostasis and downregulating c*yp19a* mRNA expression, thereby inhibiting E2 synthesis and increasing T levels [26].

## 4. Discussion

The reproductive health of aquatic organisms is an important part of the maintenance of aquatic ecosystems, especially the impact of NPs’ pollution, which has attracted increasing attention due to its ubiquity and adverse effects on aquatic ecosystems. Among the many types of microplastics, PS-NPs is a well-known and the most common chemical that affects a variety of health-related substances, including neurotoxicity and reproductive toxicity. However, the interpretation of the underlying molecular mechanisms remains limited. Our results suggest that changes in zebrafish behavior are significantly observed at higher environmental concentrations (1 mg/L) and that exposure to microplastics may lead to anxious behavior, such as increased swimming speed. This suggests that they may be disturbed by their environment and their social behavior is affected. At the same time, our study points out that zebrafish exposed to microplastics show reduced exploratory behavior, resulting in a reduced ability to adapt to new environments.

In addition, we found that long-term exposure to water-borne PS-NPs’ pollution can have certain negative effects on brain tissue and reproductive organs of fish. Exposure to PS-NPs affected both male and female zebrafish, reducing the number of sperm and oocytes in the gonads (testes and ovaries). This results in reduced spermatogenesis in male zebrafish and slower germ cell development in female zebrafish, affecting overall reproductive function. Multiple studies on lower organisms, aquatic animals, and mammals have shown that nanoparticles, especially polystyrene microbeads, exhibit reproductive toxicity to different species. For example, studies have shown that exposure to PS-NPs affects the reproductive organs and function of water fleas, rotifers, hydrops, corals, copepods, nematodes, sea urchins, mussels, zebrafish, rice crabs, oysters, and rodents [28]. This further confirms that PS-NPs produces similar tissue toxicity to the gonads [25,29].

**Figure 8:**
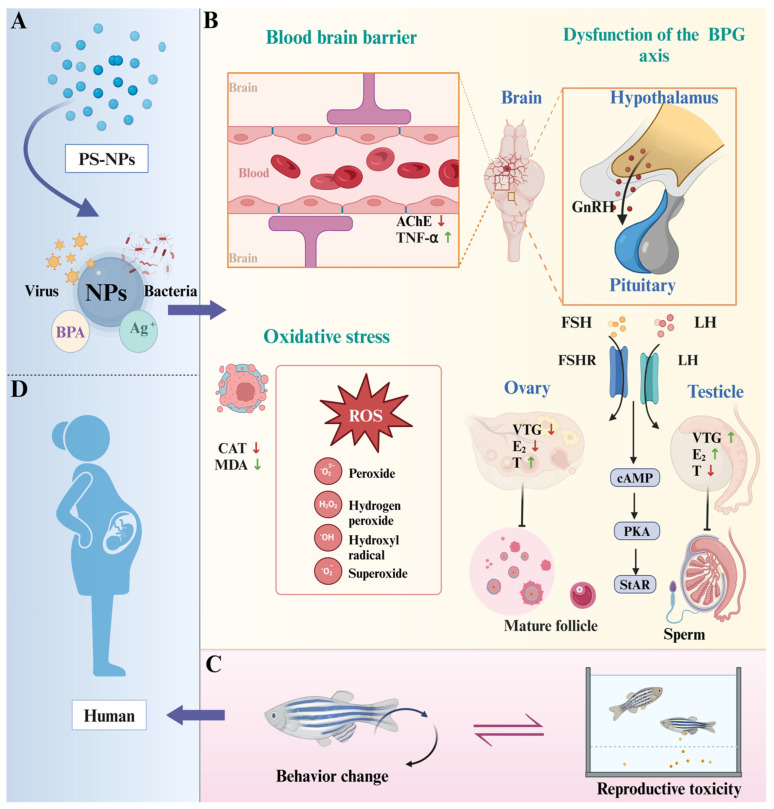
Potential mechanism for effects of PS-NPs on zebrafish neurobehavior and reproductive toxicity. (**A**) NPs carry other contaminants and bacteria and viruses [42]; (**B**) three main mechanisms; (**C**) interaction of behavioral changes and reproductive toxicity in zebrafish [11]; (**D**) potential effects on human reproduction. NPs, Nanoplastics; PS-NPs, Polystyrene nanoplastics; BPA, Bisphenol A; CAT, Catalase; MDA, Malondialdehyde; TNF-α, Tumor necrosis fac-tor-alpha; AChE, Acetylcholinesterase; E2, Estradiol; VTG, Vitellogenin; T, Testosterone; *vtg2*, Vitellogenin 2; *cyp19a*, Cytochrome P450 19A; *lhb*, Luteinizing hormone beta; *cyp17*, Cytochrome P450 17; *erα*, Estrogen receptor alpha; *vtg1*, Vitellogenin 1; *cyp19b*, Cytochrome P450 19B; *fshr*, Follicle-stimulating hormone receptor; *ar*, Androgen receptor.

In order for the reproductive organs to function properly, the redox balance between ROS production and antioxidant enzymes needs to be highly regulated and coordinated. However, when the redox system becomes unbalanced due to environmental poisons, this can lead to interference with normal physiological processes and damage to fertility. There is growing evidence that PS-NPs induces the accumulation of free radicals and increases oxidative stress, while antioxidants act as a key defense mechanism against the effects of ROS production. Similarly, this study showed that PS-NPs weakened CAT and AChE levels while increasing MDA and TNF-α concentrations in tissues [30]. These significant changes in antioxidant enzyme activity indicate oxidative damage to lipids and the destruction of O^2−^ and H_2_O_2_ balance, leading to the accumulation of free radicals in tissues [31,32]. Previous studies have found that PS-NPs induce histopathological changes in zebrafish, upregulating *gstp1*, *hsp70l*, and *ptgs2a* in the brain and liver, while downregulating *sod1* and *gpx1*a expression. This suggests that PS-NPs induce oxidative stress through ROS, altering metabolic mechanisms and inducing various toxic effects, consistent with our findings on oxidative stress levels (Figure 8B) [33]. Wang et al. discussed that decreased levels of follicle-stimulating hormones and luteinizing hormone in males might inhibit early spermatogenesis and delay the final stages of gamete maturation. Zebrafish FSHR is crucial for reproductive system function and development, triggering signaling pathways in the ovaries upon binding with FSH to regulate follicle growth, development, and maturation [34].

Under normal conditions, acetylcholinesterase (AChE) and choline acetyltransferase maintain homeostasis of acetylcholine metabolism. Sreeja Sarasamma et al. observed that neurotransmitters, including acetylcholine, play multiple roles in physiology and psychology, such as regulating heart rate, sleep, appetite, and behaviors such as emotion and fear [24]. Anxiety-like behaviors are directly related to AChE activity in the hippocampus, and NP exposure may disrupt this activity. The inhibition of AChE activity is one of the most common neurotoxic effects and is considered a reliable indicator of neurotoxicity. A reduction in AChE activity of more than 30% may interfere with the function of the cholinergic system. Studies by Minne Prust et al. have also reported the inhibition of AChE activity in fish exposed to nanoplastics. In this study, nanoparticle exposure led to uncontrolled AChE levels in zebrafish, possibly leading to the anxiety-like behavior observed [35].

In general, the reproductive process of fish is regulated by a coordinated interaction between sex steroid hormones (E2 and T) along the HPG axis and the liver. The pituitary gland secretes gonadotropins, such as FSH and LH, which bind to the gonadotropin receptor, FSHR, and LHR to control gametogenesis and synthesis of sex steroid hormones. E2 and T play a crucial role in gonad development and function. Notably, E2 is synthesized in the ovaries and then transferred to the liver to induce the production of VTG, which is critical for ovulation [26]. Therefore, assessing sex steroid hormone levels is considered to be one of the most solidifying functional endpoints of potential reproduction. Current observations show that after long-term exposure to PS-NPs, VTG levels in female zebrafish decrease after exposure to nanoparticles, while VTG levels in male zebrafish increase, indicating interference in estradiol production. Estradiol and ketotestosterone have different ups and downs according to sex. T levels in the gonads of female zebrafish increased significantly, while E2 levels decreased in a dose-dependent manner, suggesting that this may be a reasonable cause of ovarian developmental delay [36]. In male zebrafish, the expression of cyp19a decreased, while cyp19b increased. The decrease of E2 level and the increase of T level in female zebrafish are consistent with the down-regulation of cyp19a, indicating that cyp19a regulates E2 level in females. Instead, the increase in the E2 levels and the decrease in the T levels in males may be due to cyp19b, which may regulate the E2 levels in males. Taking into account the idea that steroid production is responsible for the production of sex steroid hormones, our results show that as *fshr* transcription levels increase and lhb transcription levels decrease in female zebrafish, PS-NPs’ exposure alters the expression of genes involved in steroid production. Similarly, it was observed that PS-MPs exposure down-regulated lhb mRNA expression in female zebrafish, indicating delayed oocyte maturation. Correspondingly, histological examination revealed a lack of connectivity between the follicle cell layer and oocyte cell membrane after exposure to higher concentrations of PS-MPs, confirming the idea that PS-MPs may contribute to ovarian maturation and delayed development. In addition, our data showed that microplastic exposure significantly affected gene expression in the gonads and the ovarian steroid-producing pathway. In female fish, PS-NPs caused a significant downregulation of vtg2, cyp19a, cyp19b, and lhb, while upregulating ar in the brain steroid-producing pathway. These alterations in expression may lead to an impaired synthesis and secretion of the gonadotropin-releasing hormone (GnRH), FSH, and LH, which are essential for the vitamin BPG axis. Previous studies have also reported a transcription dysregulation of steroid-producing genes after exposure to environmental pollutants, leading to the disruption of sex steroid biosynthesis and steroid production in zebrafish. In addition, mRNA expression levels of fshr and ar decreased in males. This reduction in fshr and ar levels may lead to a lower proportion of mature spermatocytes in male zebrafish exposed to PS-NPs for 21 days. Previous studies have found that the expression level of StAR mRNA and T and LH levels are significantly reduced, which may be because PS-NPs down-regulates the LHR/cAMP/PKA/STAR pathway, resulting in lower testosterone levels [37].

In addition, MPs have a higher number of interacting amino acid residues and hydrogen bonds than natural ligands, suggesting that they may have a stronger binding affinity that may lead to the competitive inhibition and functional disruption of these receptors [36,38]. PS-NPs may promote the synthesis of VTG in the liver by inducing endogenous estrogen to bind to specific adenylate (erα), thus acting as an endocrine disruptor. Therefore, the increased level of VTG in male zebrafish may be due to increased E2 synthesis [29].

In this study, multiple gene expressions were altered in PS-NPs-exposed zebrafish compared to controls. Reduced *vtg1* and *vtg2* levels decreased the nutrients and energy provided for oocyte development, affecting vitellogenesis. Increased *vtg1* levels suggest estrogen induction by PS-NPs in male zebrafish. These changes disrupt hormonal balance and reproductive physiology in zebrafish. Additionally, sex-specific differences in gene expression may result from inherent differences in sex hormone levels and detoxification abilities between males and females. At present, relevant experiments have further explored the effects of microplastics on human reproduction, and previous experiments have shown that MPs is prevalent in placenta and meconium samples, indicating widespread exposure of pregnant women and infants [39]. We speculate that microplastics can affect the pregnancy process and fetal development as endocrine disruptors, and microplastics may indirectly affect the fetus through affecting the immune system and nutrient absorption of pregnant women [40,41]. However, the specific effects require more investigation.

## 5. Conclusions

This study shows that long-term exposure to PS-NPs induces oxidative stress and hormone imbalance through the regulation of the LHR/cAMP/PKA/STAR pathway, causing hormone imbalance by inhibiting key hormone synthesis genes, thus blocking the production of steroid hormones. The delayed synthesis of steroid hormones leads to delayed gonadal development and various pathological phenomena, and eventually leads to a decrease in the number of mature oocytes and spermatocytes, which further damages the reproductive system of zebrafish. The change in the oxidative stress level caused by microplastics has a relatively obvious effect on the neurobehavior of zebrafish. Our findings support our hypothesis that microplastics have harmful effects on the brain, gonad morphology, steroid production, and BPG axis function through specific signaling pathways, providing a basis for further exploration and analyses of their reproductive toxicity mechanisms in zebrafish. However, the specific mechanism needs further study. In addition, combined with the previous experiments and the changes in neurobehavior and reproductive toxicity observed in our experiments, we believe that zebrafish behavior is highly likely to serve as an indicator of contamination-induced reproductive toxicity.

## Figures and Tables

**Table 1:** Primers used in real-time RT-PCR analysis of zebrafish gonads after 21 days of exposure to PS-NPs.

Gene	Forward Sequence	Reverse Sequence	GenBank No.
*vtg1*	TTTGGTGTGAGAACTGGAGG	TGCTAGTGGTTTATCGGTTGG	NM_001044897
*vtg2*	TTGTGGAAAGGCTGATGGAG	CAGATTCAAGTTTCATGCGGC	NM_001044913
*erα*	GTGGGTTTGGTGAGATGTTTTG	CAGTCATTTTCCCCAGTTTTCC	NM_152959
*lhb*	ATACCAACAGAACTACGAAAATTGAC	ATACCAACAGAACTACGAAAATTGAC	NM_205622
*fshr*	TGCGTTTCCCATCTTCAGTC	TGCGTTTCCCATCTTCAGTC	NM_001001812
*cyp19a*	CTACTTCAGATTGGACTGGCTG	TGGTCAAGTTTCTCTGCGTG	NM_131154
*cyp19b*	GACTACATTGATGGCTACCGG	ATGGCTGGAAGTAACGACTG	NM_131642
*cyp17*	CCTGACATTCTTATCCTACCCATC	TCGCCCTAACCTATCTAATCCC	NM_212806
*ar*	CCAAAGGTCGAAAAGGTTGTG	CTGAGCGATGCCTATAACCTG	XM_005171775

## Data Availability

The original contributions presented in the study are included in the article/Supplementary Materials, further inquiries can be directed to the corresponding author/s.

## Supplementary Materials

The following supporting information can be downloaded at: https://www.mdpi.com/article/10.3390/toxics12080617/s1, Video S1: NTT-CK.mp4; Video S2: NTT-NPs.mp4; Video S3: OFT-CK.mp4; Video S4: OFT-NPs.mp4.

## Abbreviations

PS	Polystyrene
NPs	Nanoplastics
PS-NPs	Nano-polystyrenes
PBA	Bisphenol A
MNPs	Microplastics and nanoplastics/Micro- and nanoplastics
ROS	Reactive oxygen species
BPG	Brain–Pituitary–Gonadal
NTT	The novel tank test
OFT	The open field test
HE	Hematoxylin and eosin
CAT	Catalase
MDA	Malondialdehyde
VTG	Vitellogenin
TNF-α	Tumor necrosis factor-alpha
AChE	Acetylcholinesterase
T	Testosterone
E2	Estradiol
cDNA	Complementary DNA
Sg	Spermatogonia
Sc	Spermatocytes
St	Spermatids
Sz	Spermatozoa
POs	Perinucleolar oocytes
COs	Cortical alveolar oocytes
EVs	Early vitellogenic oocytes
LVs	Late vitellogenic oocytes
ARs	Androgen receptors
LH	Luteinizing hormone
ERα	Estrogen receptor α
FSH	Follicle-stimulating hormone receptor

## References

[1] dos Santos Silva J., Cidade M.J.A., Panero F.d.S., Ribeiro L.B., Campos da Rocha F.O. (2024). Microplastic Pollution in the Amazon Basin: Current Scenario, Advances and Perspectives. Sci. Total Environ..

[2] Haward M. (2018). Plastic Pollution of the World’s Seas and Oceans as a Contemporary Challenge in Ocean Governance. Nat. Commun..

[3] Dissanayake P.D., Kim S., Sarkar B., Oleszczuk P., Sang M.K., Haque M.N., Ahn J.H., Bank M.S., Ok Y.S. (2022). Effects of Microplastics on the Terrestrial Environment: A Critical Review. Environ. Res..

[4] Bradney L., Wijesekara H., Palansooriya K.N., Obadamudalige N., Bolan N.S., Ok Y.S., Rinklebe J., Kim K.-H., Kirkham M.B. (2019). Particulate Plastics as a Vector for Toxic Trace-Element Uptake by Aquatic and Terrestrial Organisms and Human Health Risk. Environ. Int..

[5] Bermúdez J.R., Swarzenski P.W. (2021). A Microplastic Size Classification Scheme Aligned with Universal Plankton Survey Methods. MethodsX.

[6] Cao F., Zhu L., Li H., Yu S., Wang C., Qiu L. (2016). Reproductive Toxicity of Azoxystrobin to Adult Zebrafish (*Danio rerio*). Environ. Pollut..

[7] Chen J., Liang Q., Zheng Y., Lei Y., Gan X., Mei H., Bai C., Wang H., Ju J., Dong Q. (2024). Polystyrene Nanoplastics Induced Size-Dependent Developmental and Neurobehavioral Toxicities in Embryonic and Juvenile Zebrafish. Aquat. Toxicol..

[8] Yi J., Ma Y., Ma J., Yu H., Zhang K., Jin L., Yang Q., Sun D., Wu D. (2023). Rapid Assessment of Ocular Toxicity from Environmental Contaminants Based on Visually Mediated Zebrafish Behavior Studies. Toxics.

[9] Ziajahromi S., Kumar A., Neale P.A., Leusch F.D.L. (2017). Impact of Microplastic Beads and Fibers on Waterflea (*Ceriodaphnia dubia*) Survival, Growth, and Reproduction: Implications of Single and Mixture Exposures. Environ. Sci. Technol..

[10] Li J., Liu J., Wang X., Zhang T., Wang D., Shan E., Teng J., Zhao J., Wang Q. (2024). Vertical Transfer of Microplastics in Nearshore Water by Cultured Filter-Feeding Oysters. J. Hazard. Mater..

[11] Wu B., Yu H., Yi J., Lei P., He J., Ruan J., Xu P., Tao R., Jin L., Wu W. (2024). Behavioral Studies of Zebrafish Reveal a New Perspective on the Reproductive Toxicity of Micro- and Nanoplastics. Toxics.

[12] Howe K., Clark M.D., Torroja C.F., Torrance J., Berthelot C., Muffato M., Collins J.E., Humphray S., McLaren K., Matthews L. (2013). The Zebrafish Reference Genome Sequence and Its Relationship to the Human Genome. Nature.

[13] Wang L., Liu F., Fang Y., Ma J., Wang J., Qu L., Yang Q., Wu W., Jin L., Sun D. (2023). Advances in Zebrafish as a Comprehensive Model of Mental Disorders. Depress. Anxiety.

[14] Tsang B., Zahid H., Ansari R., Lee R.C.-Y., Partap A., Gerlai R. (2017). Breeding Zebrafish: A Review of Different Methods and a Discussion on Standardization. Zebrafish.

[15] Martínez-Álvarez I., Le Menach K., Cajaraville M.P., Budzinski H., Orbea A. (2024). Effects of Polystyrene Nano- and Microplastics and of Microplastics with Sorbed Polycyclic Aromatic Hydrocarbons in Adult Zebrafish. Sci. Total Environ..

[16] Shi C., Liu Z., Yu B., Zhang Y., Yang H., Han Y., Wang B., Liu Z., Zhang H. (2024). Emergence of Nanoplastics in the Aquatic Environment and Possible Impacts on Aquatic Organisms. Sci. Total Environ..

[17] Alix M., Kjesbu O.S., Anderson K.C. (2020). From Gametogenesis to Spawning: How Climate-Driven Warming Affects Teleost Reproductive Biology. J. Fish Biol..

[18] Servili A., Canario A.V.M., Mouchel O., Muñoz-Cueto J.A. (2020). Climate Change Impacts on Fish Reproduction Are Mediated at Multiple Levels of the Brain-Pituitary-Gonad Axis. Gen. Comp. Endocrinol..

[19] Zohar Y. (2021). Fish Reproductive Biology—Reflecting on Five Decades of Fundamental and Translational Research. Gen. Comp. Endocrinol..

[20] Borella M.I., Chehade C., Costa F.G., de Jesus L.W.O., Cassel M., Batlouni S.R., Baldisserotto B., Urbinati E.C., Cyrino J.E.P. (2020). Chapter 14—The Brain-Pituitary-Gonad Axis and the Gametogenesis. Biology and Physiology of Freshwater Neotropical Fish.

[21] Lu X., Yu R.M.K., Murphy M.B., Lau K., Wu R.S.S. (2014). Hypoxia Disrupts Gene Modulation along the Brain-Pituitary-Gonad (BPG)-Liver Axis. Ecotoxicol. Environ. Saf..

[22] Zhou W., Tong D., Tian D., Yu Y., Huang L., Zhang W., Yu Y., Lu L., Zhang X., Pan W. (2023). Exposure to Polystyrene Nanoplastics Led to Learning and Memory Deficits in Zebrafish by Inducing Oxidative Damage and Aggravating Brain Aging. Adv. Health Mater..

[23] Westerfield M. (1995). The Zebrafish Book: A Guide for the Laboratory Use of Zebrafish (Danio rerio).

[24] Sarasamma S., Audira G., Siregar P., Malhotra N., Lai Y.-H., Liang S.-T., Chen J.-R., Chen K.H.-C., Hsiao C.-D. (2020). Nanoplastics Cause Neurobehavioral Impairments, Reproductive and Oxidative Damages, and Biomarker Responses in Zebrafish: Throwing up Alarms of Wide Spread Health Risk of Exposure. Int. J. Mol. Sci..

[25] Qiang L., Cheng J. (2021). Exposure to Polystyrene Microplastics Impairs Gonads of Zebrafish (*Danio rerio*). Chemosphere.

[26] Chatterjee A., Maity S., Banerjee S., Dutta S., Adhikari M., Guchhait R., Biswas C., De S., Pramanick K. (2022). Toxicological impacts of nanopolystyrene on zebrafish oocyte with insight into the mechanism of action *Sci*. Total Environ..

[27] Zhang Y., Yang Y., Tao Y., Guo X., Cui Y., Li Z. (2023). Phthalates (PAEs) and Reproductive Toxicity: Hypothalamic-Pituitary-Gonadal (HPG) Axis Aspects. J. Hazard. Mater..

[28] Yan Z., Zhao H., Zhu P., Wang Y., Hou J., Lu G., He C. (2024). Polystyrene Microplastics Alter the Trophic Transfer and Biotoxicity of Fluoxetine in an Aquatic Food Chain. J. Hazard. Mater..

[29] Lin X., Wang Y., Yang X., Watson P., Yang F., Liu H. (2023). Endocrine Disrupting Effect and Reproductive Toxicity of the Separate Exposure and Co-Exposure of Nano-Polystyrene and Diethylstilbestrol to Zebrafish. Sci. Total Environ..

[30] Shi X., Xu T., Gao M., Bi Y., Wang J., Yin Y., Xu S. (2024). Combined Exposure of Emamectin Benzoate and Microplastics Induces Tight Junction Disorder, Immune Disorder and Inflammation in Carp Midgut via Lysosome/ROS/Ferroptosis Pathway. Water Res..

[31] Lei P., Zhang W., Ma J., Xia Y., Yu H., Du J., Fang Y., Wang L., Zhang K., Jin L. (2023). Advances in the Utilization of Zebrafish for Assessing and Understanding the Mechanisms of Nano-/Microparticles Toxicity in Water. Toxics.

[32] Gou X., Fu Y., Li J., Xiang J., Yang M., Zhang Y. (2024). Impact of Nanoplastics on Alzheimer’s Disease: Enhanced Amyloid-β Peptide Aggregation and Augmented Neurotoxicity. J. Hazard. Mater..

[33] Wang Y., Li Y., Chen Q., Liu Z. (2019). Long-Term Exposure of Xenoestrogens with Environmental Relevant Concentrations Disrupted Spermatogenesis of Zebrafish through Altering Sex Hormone Balance, Stimulating Germ Cell Proliferation, Meiosis and Enhancing Apoptosis. Environ. Pollut..

[34] Wang Y., Li Y., Chen Q., Liu Z. (2018). Diethylstilbestrol Impaired Oogenesis of Yellow Catfish Juveniles through Disrupting Hypothalamic-Pituitary-Gonadal Axis and Germ Cell Development. J. Appl. Toxicol..

[35] Prüst M., Meijer J., Westerink R.H.S. (2020). The Plastic Brain: Neurotoxicity of Micro- and Nanoplastics. Part. Fibre Toxicol..

[36] Gupta P., Mahapatra A., Suman A., Ray S.S., Malafaia G., Singh R.K. (2023). Polystyrene Microplastics Disrupt Female Reproductive Health and Fertility via Sirt1 Modulation in Zebrafish (*Danio rerio*). J. Hazard. Mater..

[37] Larsen M.G., Baatrup E. (2010). Functional Behavior and Reproduction in Androgenic Sex Reversed Zebrafish (*Danio rerio*). Environ. Toxicol. Chem..

[38] Ullah S., Ahmad S., Guo X., Ullah S., Ullah S., Nabi G., Wanghe K. (2023). A Review of the Endocrine Disrupting Effects of Micro and Nano Plastic and Their Associated Chemicals in Mammals. Front. Endocrinol..

[39] Liu S., Liu X., Guo J., Yang R., Wang H., Sun Y., Chen B., Dong R. (2023). The Association between Microplastics and Microbiota in Placentas and Meconium: The First Evidence in Humans. Environ. Sci Technol..

[40] Liu S., Guo J., Liu X., Yang R., Wang H., Sun Y., Chen B., Dong R. (2023). Detection of Various Microplastics in Placentas, Meconium, Infant Feces, Breastmilk and Infant Formula: A Pilot Prospective Study. Sci. Total Environ..

[41] Paul I., Mondal P., Haldar D., Halder G. (2024). Beyond the Cradle—Amidst Microplastics and the Ongoing Peril during Pregnancy and Neonatal Stages: A Holistic Review. J. Hazard. Mater..

[42] Lomonaco T., Manco E., Corti A., La Nasa J., Ghimenti S., Biagini D., Di Francesco F., Modugno F., Ceccarini A., Fuoco R. (2020). Release of Harmful Volatile Organic Compounds (VOCs) from Photo-Degraded Plastic Debris: A Neglected Source of Environmental Pollution. J. Hazard. Mater..

